# Harnessing snake venom cardiotoxins for antimicrobial peptide discovery

**DOI:** 10.1038/s44386-026-00073-2

**Published:** 2026-09-16

**Authors:** Bruno Mendes, José R. Almeida, Valeria Castelletto, Ian W. Hamley, Glyn Barrett

**Affiliations:** 1 School of Biological Sciences, University of Reading, Reading, UK; 2 School of Chemistry, Food and Pharmacy, University of Reading, Reading, UK

**Keywords:** Biochemistry, Biotechnology, Computational biology and bioinformatics, Drug discovery, Microbiology

## Abstract

Snake venoms are a rich source of bioactive molecules with considerable potential for drug discovery. Cardiotoxins (CTXs) from cobras (*Naja* spp.) are membrane-active venom proteins and promising templates for antimicrobial peptide development. Here, we combined two computational toxin-mining strategies to identify antimicrobial peptide candidates from CTXs. First, AMPA-guided sequence mining was used to detect encrypted antimicrobial regions within CTX sequences. Second, sequence alignment and consensus-sequence analysis were employed to generate peptides from a conserved CTX scaffold. Candidate peptides were prioritised using multiple machine learning- and deep learning-based antimicrobial peptide prediction tools, resulting in fourteen CTX-inspired peptides, including two modified derivatives of CTX-p5. Antimicrobial screening showed limited activity across the peptide panel, with CTX-p5 exhibiting the strongest activity against both Gram-negative and Gram-positive bacteria. Neither truncation nor chemical modification improved its antibacterial potency. Mechanistic studies, including atomic force microscopy, flow cytometry, fluorescence microscopy and membrane permeabilisation assays, indicated that CTX-p5 disrupts bacterial membrane integrity. These findings identify CTXs as a source of antimicrobial peptide templates while highlighting the limitations of current computational prioritisation approaches and the need for more accurate toxin-mining workflows to enhance peptide selection during toxin-inspired drug discovery.

## Introduction

Snake venoms constitute a natural library of bioactive chemical startpoints with considerable potential for biomedical and biotechnological applications, including the discovery of novel anti-infective agents such as antibiotics^1,2^. Components of venom have evolved to interact with biological targets such as cell membranes, ion channels, and enzymatic pathways, many of which are also present in microorganisms^3^. In addition, venom glands express proteins with antimicrobial activity that are hypothesised to provide some protection to snakes from microbial infections associated with prey capture and venom storage^4^. This evolutionary refinement makes snake venom peptides and proteins particularly attractive scaffolds for antimicrobial discovery, as they often display potent membrane-disrupting, enzymatic, or immunomodulatory activities that can be harnessed to combat microbes, especially pathogenic bacteria^5,6^. Importantly, the pharmaceutical market already includes venom-derived molecules that have successfully overcome translational barriers, demonstrating the therapeutic potential of venom components as useful templates in biopharmaceutical development^7^.

Despite this promise, the discovery of venom-derived bioactive compounds has traditionally relied on labour-intensive workflows involving venom extraction, chromatographic fractionation or recombinant production and functional screening of individual components^8,9^. While such approaches have led to important advances, they remain time-consuming and resource-intensive, particularly given the significant diversity of venomous snake species and the biochemical complexity of their venoms. Another challenge involves the limited translational potential of large biomolecules and the difficulties associated with their large-scale production^5^. Recent advances in ‘omics tools have expanded the catalogue of venom toxin protein sequences available in public databases, such as Sanke Venom Database (https://www.snakevenomdb.org/) and Venom Zone (https://venomzone.expasy.org/), revealing largely unexplored molecular diversity^10–12^. These datasets now offer an alternative route for discovery, enabling the exploration of venom repertoires directly at the sequence level.

One promising strategy emerging from this protein sequence information is the design of toxin-inspired peptides, in which short peptide fragments or engineered sequences are derived from known toxin scaffolds^13^. Rather than isolating entire toxins from venoms, this innovative approach aims to reproduce key structural or physicochemical features responsible for biological activity, such as amphipathicity, charge distribution, or membrane-interacting motifs^14^. In some cases, bioactive peptides may already be embedded within larger protein sequences as encrypted peptides, which remain inactive in the context of the parent protein but can exhibit biological activity when released as shorter fragments^15^. Such strategies allow researchers to capture the functional essence of complex toxins while avoiding many of the translational limitations associated with larger venom proteins. The identification of such hidden functional motifs within toxin sequences represents an emerging concept in peptide discovery and provides an additional opportunity to mine venom proteins for antimicrobial candidates^16,17^. However, despite the growing availability of toxin sequence data, relatively few studies have explored the in silico extraction of functional peptide fragments from toxin architectures as a route for antimicrobial discovery^18,19^. This approach is particularly relevant for membrane-active toxins, including snake venom CTXs from cobras of the genus *Naja*.

CTXs belong to the three-finger toxin (3FTx) superfamily and are small, highly stable, basic polypeptide toxins of approximately 60 amino acids stabilised by four conserved disulfide bonds^20^. Structurally, they adopt the characteristic three-finger fold composed of three β-sheet–rich loops extending from a compact disulfide-stabilised core^21^. CTXs are typically cationic molecules, with isoelectric points often above 9, and display pronounced amphipathic surfaces that favour interactions with lipid membranes^22^. These physicochemical properties underpin their strong affinity for phospholipid bilayers, enabling them to insert into membranes, disrupt lipid organisation, and induce pore formation or membrane destabilisation^23^. Cardiotoxins are also among the most abundant components in many *Naja* venoms, in some cases representing a substantial proportion of the total venom proteome^24^. Although originally characterised for their cytotoxic and cardiotoxic effects, CTXs exhibit a broad spectrum of biological activities, including cytolytic, antimicrobial and immunomodulatory effects, largely driven by their membrane-active nature^22^. The combination of their structural stability, membrane affinity and relatively short sequence length makes CTXs attractive molecular templates for the identification of shorter functional fragments with antimicrobial potential. Consequently, rational mining of CTX sequences offers a promising route to identify peptide motifs that retain membrane-disrupting activity while providing novel starting points for the development of novel antimicrobial agents. In the same lines, related cardiotoxin-like basic proteins (CLBPs), which are generally longer and may display reduced membranolytic action compared with CTXs, are also present within some cobra venoms and have not been intensively explored for antibiotic discovery.

Recent developments in artificial intelligence (AI) provide new opportunities to potentiate this process^25^. Computational tools capable of predicting antimicrobial potential, structural propensity, and physicochemical properties from amino acid sequences now enable rapid prioritisation of candidate peptides before synthesis and experimental testing^26^. Emerging approaches that combine large-scale venomics datasets with AI-driven analysis have begun to reveal that venom proteins can harbour encrypted peptide motifs^18^. Hence, integrating toxin sequence mining with AI-guided prediction may offer a strategy to identify toxin-inspired bioactive peptides. In the present study, we applied two computational toxin-mining strategies combined with machine learning/deep learning-based antimicrobial peptide predictions to analyse CTX sequences from *Naja* species and identify molecular fragments with predicted antimicrobial properties. The resulting CTX-inspired peptides were structurally characterised and experimentally evaluated for antimicrobial activity, membrane-disrupting properties, and haemolytic effects, providing insight into both the potential and limitations of computationally guided AMP predictions.

## Results

### Computational analysis of Naja CTXs reveals antimicrobial peptide (AMP) motifs

**Fig. 1:**
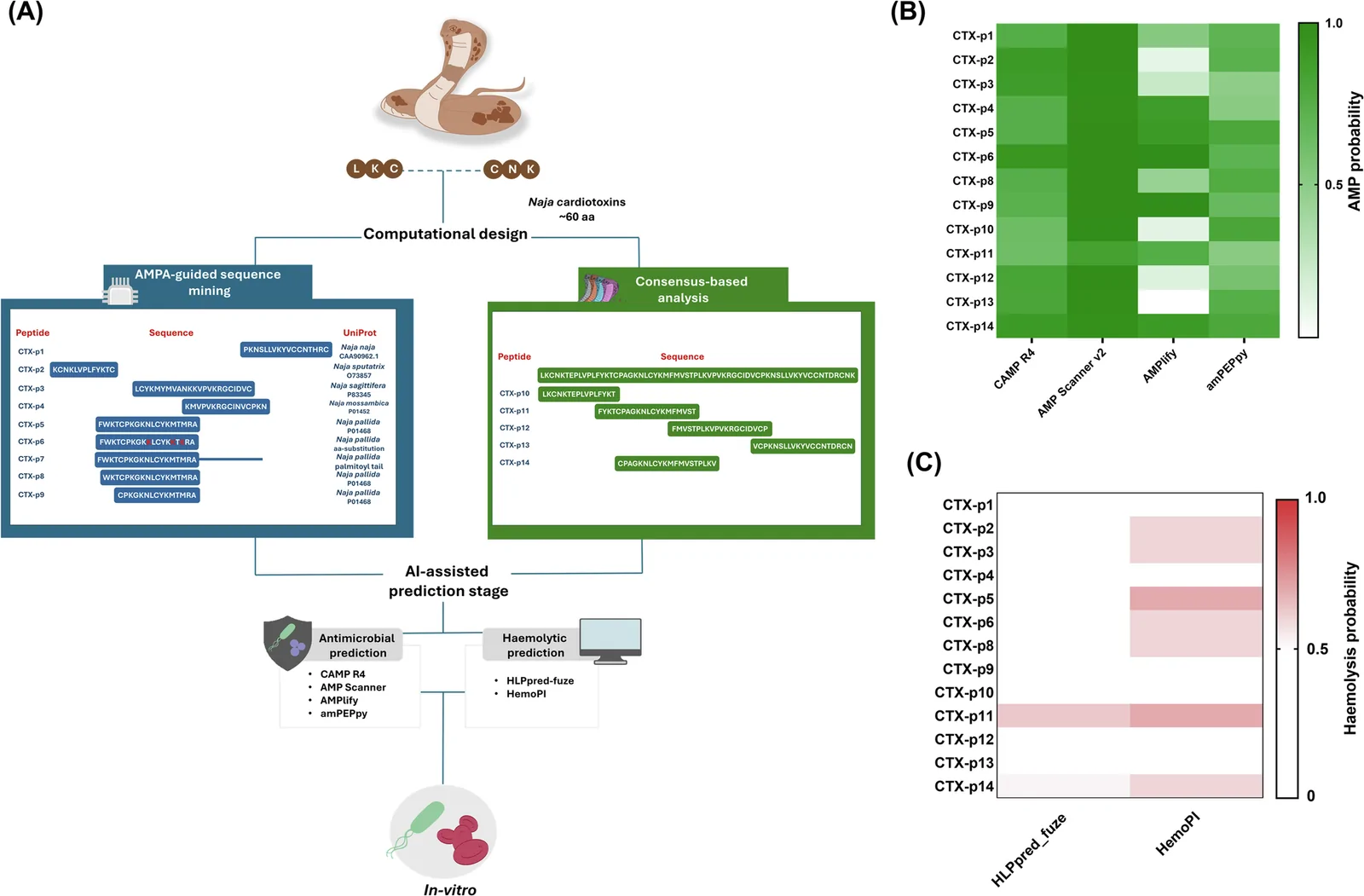
Schematic bioprospecting workflow for the discovery and prediction of antimicrobial peptides from *Naja* CTXs. **A** Pipeline for mining CTXs from *Naja* spp. using two complementary strategies. Blue box represents an approach based on the AMPA algorithm, which identified seven antimicrobial stretches derived from known CTX protein families: CTX-p1, CTX-p2, CTX-p3, CTX-p4, CTX-p5, CTX-p8, and CTX-p9 from naturally occurring sequences. In addition, CTX-p6 and CTX-p7 were designed based on CTX-p5, incorporating amino acid substitutions and a palmitoyl tail. The green box represents consensus-derived regions (CTX-p10 to CTX-p14), identified from sequence alignment across 92 entries from UniProtKB database. All candidate peptides were subsequently evaluated by in silico analyses to predict antimicrobial activity and haemolytic potential, followed by wet lab prediction against bacterial strains and red blood cells. **B**, **C** Computational prediction for antimicrobial activity and haemolytic properties, respectively. Prediction outputs generated by the individual antimicrobial prediction platforms were interpreted independently according to the scoring systems and thresholds defined by each algorithm. Scores were not normalised. Heatmaps was used to qualitatively assess agreement among predictors.

To explore the potential of snake venom CTXs as templates for AMP design, CTX sequences from *Naja* species were retrieved from the NCBI protein database and analysed for sequence similarity. Multiple sequence alignment revealed a high level of conservation across the analysed CTXs, indicating that key structural and functional motifs are preserved within this toxin family. Based on these findings, two complementary approaches were employed to identify candidate CTX-derived peptide fragments, which were subsequently prioritised using machine learning/deep learning-based AMP prediction tools (Fig. 1). In general, peptide generation was based on AMPA-guided sequence mining and rational extraction of toxin-derived regions or consensus-sequence approach, whereas machine learning/deep learning-based AMP prediction tools were used for candidate in silico analysis and prioritisation.

In the first approach (blue box, deterministic physicochemical-property prediction; Fig. 1A), CTX sequences from eight *Naja* species were selected based on high similarity to *Naja atra* (UniProt: P60304), a toxin previously used as a template for engineered antimicrobial peptides following sequence optimisation and amino acid substitutions that enhanced antibacterial activity^27^. Sequence alignment revealed a high degree of conservation across the toxins, with several regions enriched in cationic and hydrophobic residues, hallmarks of membrane-active antimicrobial peptides (AMPs) (Figs. S1A and S1B). To identify specific antimicrobial motifs, aligned sequences were analysed using the AMPA algorithm^28^, which revealed bioactive antimicrobial stretches within protein sequences (Fig. S1C). This analysis identified seven putative antimicrobial regions based on AMPA scores and positional conservation. Candidates were subsequently assessed using multiple antimicrobial prediction tools, including AMPlify^29^, CAMP-R4^30^, amPEPpy^31^ and Antimicrobial Peptide Scanner v2^32^, alongside haemolytic prediction platforms such as HemoPI^33^ and HLPpred-Fuse^34^, confirming their potential as antimicrobial candidates. This computational prioritisation strategy resulted in nine candidate peptides each with unique sequences (CTX-p1–CTX-p9), along with two rationally designed derivatives based on CTX-p5: CTX-p6, which incorporates amino acid substitutions to increase cationicity, and CTX-p7, modified with a palmitoyl tail to enhance membrane interaction.

In the second strategy (green box, consensus-based; Fig. 1A), five additional peptides (CTX-p10–CTX-p14) were generated based on a consensus CTX sequence derived from 92 entries retrieved from UniProtKB (Figure. S2). All peptides, with the exception of the chemically modified lipidated analogue, were subsequently evaluated using four independent AI-based antimicrobial prediction tools, including CAMP-R4, AMP Scanner v2, AMPlify and AMPEPpy to assess their antibacterial potential. The predictions are summarised in a heatmap (Fig. 1B, C), where darker green colours indicate higher predicted antimicrobial activity. Overall, most peptides exhibited high antimicrobial probability scores across at least two algorithms, supporting their selection for subsequent in vitro validation. However, variability between predictors was observed, with some peptides receiving strong scores from certain models but lower scores from others, reflecting differences in algorithm training and prediction criteria. Finally, physicochemical properties were assessed using the Database of Antimicrobial Activity and Structure of Peptides (DBAASP)^35^ (Fig. S3). CTX-p6 and CTX-p14 emerged as the most promising antibacterial candidates, followed by CTX-p5 and CTX-p9, which share similar sequence-derived properties. In contrast, CTX-p11 displayed a high aggregation propensity, suggesting potential limitations in stability or increased toxicity risk. Taken together, 14 peptides were chemically synthesised for experimental evaluation. Analytical characterisation confirmed the successful production of all designed peptides, with high-performance liquid chromatography indicating a purity greater than 95% for each peptide. The identity of the peptides was further verified by mass spectrometry, which confirmed the expected molecular masses corresponding to their predicted sequences (Fig. S5).

### CTX-inspired peptides display distinct structural responses in membrane-mimicking environments

**Fig. 2:**
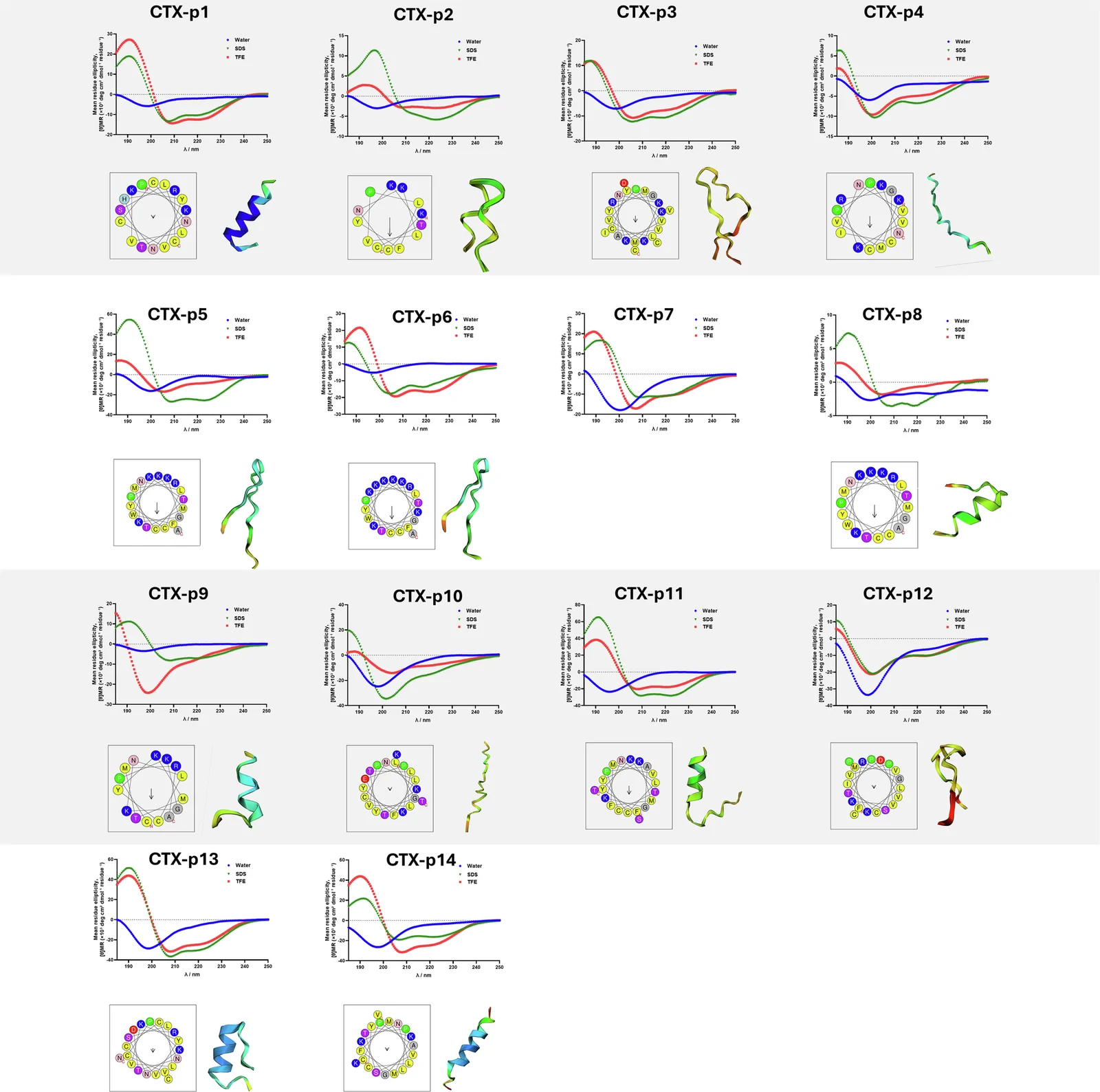
Structural analysis reveals heterogeneous conformational behaviour among CTX-derived peptides. Circular dichroism (CD) spectra were recorded for each peptide in aqueous solution and in membrane-mimicking environments using sodium dodecyl sulfate (SDS) and trifluoroethanol (TFE). CD measurements were performed using a Chirascan spectropolarimeter to evaluate environment-dependent conformational changes. For each peptide, the upper panel shows the CD spectra obtained under the three conditions (water, SDS, and TFE). The lower panels present the corresponding helical wheel projections and structural models predicted using AlphaFold. Structural models are coloured according to the AlphaFold predicted local distance difference test (pLDDT) confidence score, where blue indicates higher confidence and orange/red lower confidence regions. Helical wheel projections and structural characterisation using AlphaFold were not performed for CTX-p7 due to software limitations in modelling chemically modified (lipidated) peptides. Helical wheel projections represent theoretical amphipathic residue distributions assuming an idealised α-helical conformation and are intended only to illustrate residue segregation. They are independent of the AlphaFold structural predictions and should not be interpreted as predicted peptide conformations. Some peptides are predicted by AlphaFold to adopt partially folded or β-hairpin-like conformations, suggesting that amphipathicity is not restricted to α-helical structures. CD spectra are presented as mean residue ellipticity (MRE; [θ]MR), expressed in units of ×10³ deg cm² dmol⁻¹ residue⁻¹.

Structural analysis of the CTX-inspired peptides revealed marked heterogeneity across the peptide set. AlphaFold modelling predicted partial helical propensity for several peptides, including CTX-p1, CTX-p8, CTX-p9, CTX-p11, CTX-p13 and CTX-p14, although the reliability of such predictions for short flexible peptides remains limited. Helical wheel projections were used to illustrate the theoretical amphipathic distribution of residues assuming an idealised α-helical conformation. Some peptides were predicted by AlphaFold to adopt partially folded or β-hairpin-like conformations rather than continuous α-helices (Fig. 2). Therefore, these theoretical projections did not necessarily correlate with experimentally observed α-helical conformations.

Experimental circular dichroism analysis likewise revealed diverse conformational behaviour. In aqueous solution, most peptides displayed spectra characteristic of predominantly disordered structures. In contrast, under membrane-mimicking conditions, a subset of peptides showed increased structural ordering. In particular, CTX-p1, CTX-p8, CTX-p11, CTX-p13 and CTX-p14 exhibited the characteristic α-helical CD signature, with two negative bands near 208 and 222 nm, indicating helix formation in the presence of SDS or TFE. Other peptides showed only partial structural induction or largely unordered profiles (Fig. 2). DichroWebGit deconvolution analysis further supported heterogeneous conformational behaviour across the peptide set, suggesting inducible helical propensity for only a subset of peptides under SDS and TFE conditions (Figure. S4). Overall, these findings indicate partial agreement between AlphaFold-predicted structural propensity and experimental observed conformational behaviour under membrane-mimicking conditions, suggesting that only a subset of CTX-inspired peptides displays inducible helical character. However, membrane disruption by antimicrobial peptides does not necessarily require stable α-helical conformations and may involve alternative membrane interaction mechanisms.

### CTX-p5 exhibits antibacterial and membranolytic activity, whereas most computationally prioritised CTX-derived peptides display limited potency

**Fig. 3:**
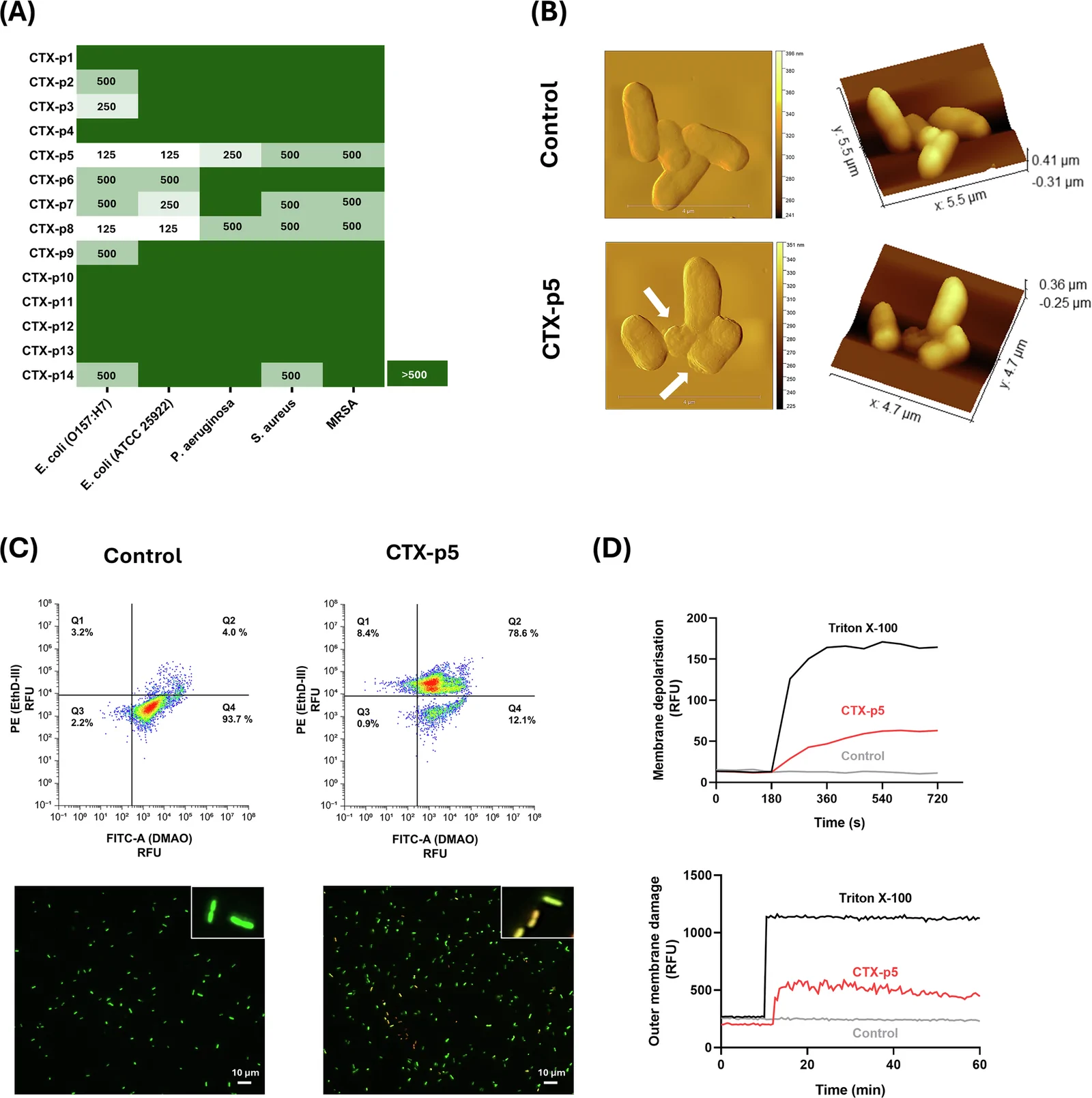
Experimental screening reveals limited potency of computationally prioritised peptides while CTX-p5 disrupts bacterial membranes. Antibacterial efficacy and membrane interaction analysis of CTX-inspired peptides. **A** Antimicrobial screening of the designed peptides against Gram-negative and Gram-positive bacteria, including *E. coli* (ATCC 25922 and enterohaemorrhagic O157:H7), *P. aeruginosa* (PAO1) *S. aureus* (ATCC 12600) and methicillin-resistant *S. aureus* (MRSA), using broth microdilution assays to determine minimum inhibitory concentrations (MICs). **B** Morphological analysis of bacterial cells following peptide exposure using AFM. Mid-log phase *E. coli* cells were incubated with peptides and subsequently imaged to evaluate structural alterations at the cell surface. Treated cells displayed altered surface morphology and and irregular topographical features (arrows) compared with untreated controls. **C** Assessment of bacterial membrane integrity by fluorescence microscopy and flow cytometry using live/dead staining dyes. Cells were incubated with peptide and stained with membrane-impermeable nucleic acid probes to detect compromised membranes. *X*-axis: green fluorescence intensity (DMAO; viable cells); *Y*-axis: red fluorescence intensity (EthD-III; membrane-compromised cells), both shown in relative florescence units. **D** Evaluation of peptide-induced membrane perturbation using fluorescence-based assays. Outer membrane permeability was assessed using the NPN uptake assay, while inner membrane depolarisation was monitored using the membrane potential-sensitive dye DiSC₃(5).

The antimicrobial activity of the 14 CTX-inspired peptides was evaluated against a panel of Gram-negative and Gram-positive bacteria, including *Escherichia coli* (ATCC 25922 and enterohaemorrhagic O157:H7 str. EDL933), *Pseudomonas aeruginosa* (PAO1)), *Staphylococcus aureus* (ATCC 12600) and methicillin-resistant *S. aureus* (NCTC 13656). Overall, the peptide library exhibited modest antibacterial activity, with MIC values ranging between 125 and 500 µM, while several candidates, mainly those peptides derived from the consensus-based strategy, did not display measurable activity within the tested concentration range (Fig. 3A).

Among all candidates, CTX-p5 displayed the most consistent antibacterial activity across the tested bacterial strains, with MIC values ranging between 125 and 500 µM. In line with this, peptides derived from the same CTX region, including shorter or modified variants such as CTX-p6, CTX-p7, CTX-p8 and CTX-p9 showed reduced antibacterial activity and generally exhibited higher MIC values. Given its comparatively higher potency, CTX-p5 was selected for mechanistic investigation.

Microscopy-based analyses provide initial evidence of bacterial membrane damage induced by CTX-p5. Atomic force microscopy revealed alterations in the surface morphology of *E. coli* cells following CTX-p5 treatment, including irregular cell surface features consistent with membrane perturbation (Fig. 3B). Fluorescence microscopy revealed increased uptake of membrane-impermeable dyes in peptide-treated bacterial cells compared with untreated controls, suggesting membrane-damaging activity (Fig. 3C). Flow cytometry further supported these findings by showing a marked increase in the proportion of membrane-compromised bacterial cells following exposure to CTX-p5 compared with untreated controls. Fluorescence-based live/dead staining revealed a shift toward permeabilised bacterial populations, indicating loss of membrane integrity (Fig. 3C).

The membrane-disrupting activity of CTX-p5 was further confirmed through assays measuring outer membrane permeability and inner membrane depolarisation (Fig. 3D). In the untreated control samples, fluorescence signals remained low throughout the experiment, indicating intact bacterial membranes. In contrast, treatment with Triton X-100, used as a positive control for membrane disruption, resulted in a strong and rapid increase in fluorescence, reflecting extensive membrane damage. Exposure to CTX-p5 produced a clear increase in fluorescence compared with the untreated control in both assays. In the NPN assay, the increase in fluorescence indicated enhanced permeability of the outer membrane, consistent with disruption of the lipid barrier. Similarly, the DiSC₃(5) assay revealed a marked rise in fluorescence following CTX-p5 treatment, demonstrating rapid perturbation and depolarisation of the inner membrane. Although the effect was less pronounced than that observed with Triton X-100, these results indicate that CTX-p5 compromises bacterial membrane integrity and supports a membranolytic mode of action. Taken together, these results indicate that, although most computationally prioritised CTX-derived peptides showed limited antibacterial potency, CTX-p5 exhibited moderate antimicrobial activity, likely mediated by disruption of bacterial membrane integrity.

### CTX-derived peptides show limited haemolytic activity

**Fig. 4:**
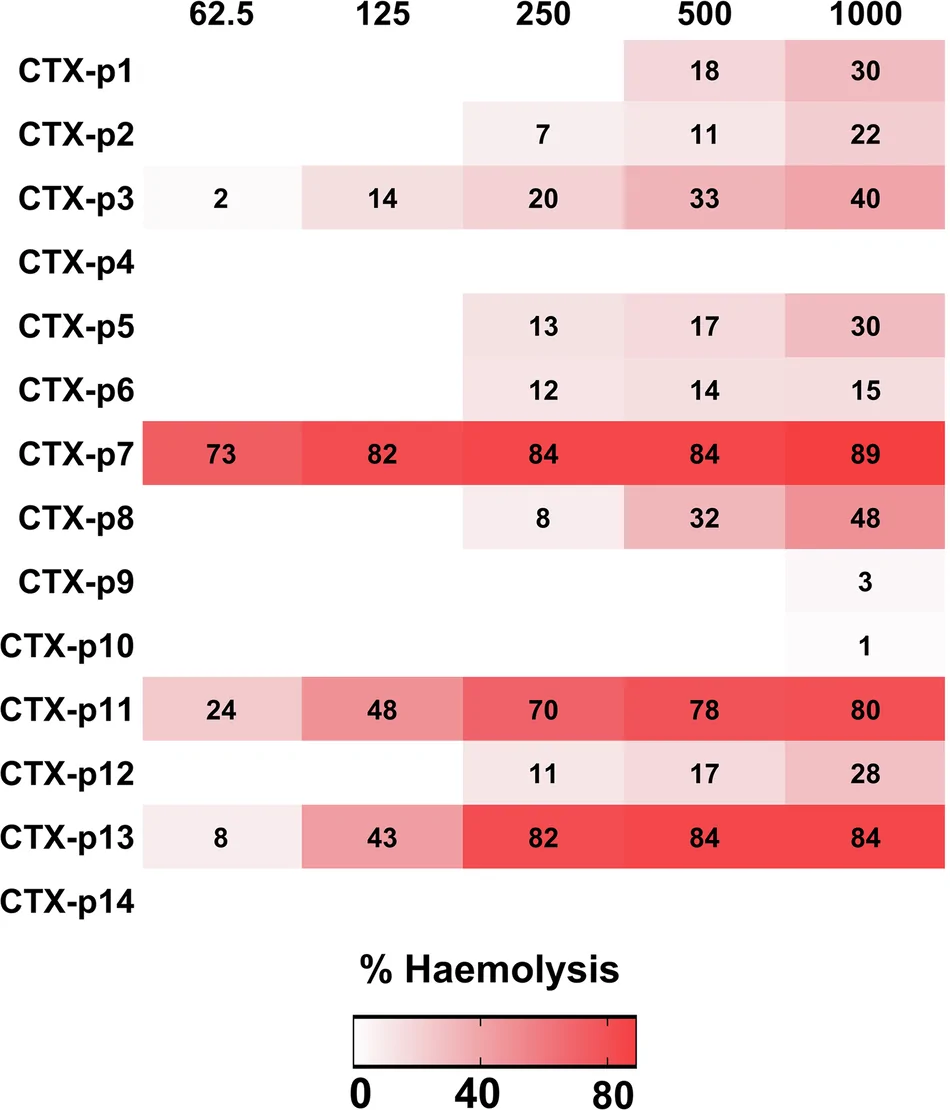
Differential haemolytic activity among CTX-derived peptides. Erythrocytes were incubated with peptides at 62.5, 125, 250, 500, and 1000 µM for 1 h at 37 °C, and haemoglobin release was quantified by measuring absorbance at 414 nm. PBS and 1% Triton X-100 served as negative and positive controls, respectively.

The haemolytic potential of the CTX-inspired peptides was evaluated using donated human red blood cells (hRBCs; erythrocytes) across concentrations ranging from 62.5 to 1000 µM (Figs. 4 and S6). Overall, the CTX-inspired peptide library exhibited variable haemolytic profiles, with the majority of CTX-derived encrypted peptides producing little or no detectable haemolysis at the lowest concentrations and increasing activity observed at higher concentrations. Among the peptides tested, CTX-p7, corresponding to the lipidated derivative of CTX-p5, displayed the highest haemolytic activity across the tested concentration range. This peptide induced a pronounced increase in haemoglobin release, particularly at concentrations of 500–1000 µM, indicating stronger disruption of erythrocyte membranes compared with the other candidates. In contrast, the non-lipidated peptide CTX-p5 produced substantially lower haemolytic effects, even at the highest tested concentrations. Most of the remaining peptides exhibited limited haemolytic activity at lower concentrations, with haemolysis becoming detectable only at higher peptide levels. This pattern suggests that the CTX-derived peptides generally display moderate to low toxicity toward erythrocytes within the tested range.

## Discussion

Membrane-active toxins represent an attractive source of molecular scaffolds for AMP discovery^36^. Many venom-derived molecules interact directly with biological membranes, a property that can be exploited for targeting bacterial cells, as the membrane is an essential and highly conserved structure whose disruption often leads to rapid cell death and reduced likelihood of resistance development^37^. Among snake venom toxins, cardiotoxins and cardiotoxin-like proteins from the genus *Naja* are well known for their affinity for lipid bilayers and their ability to perturb membrane integrity^38^. In addition to their cytotoxic properties, several studies have reported antimicrobial activity associated with cardiotoxin-derived sequences^27,39,40^. For example, rationally designed and optimised peptides derived from a cardiotoxin of *Naja atra* have been shown to reproduce the antibacterial activity of the parent protein through membrane disruption^27^, highlighting the potential of cardiotoxin scaffolds as useful templates for AMP design. These observations suggest that cardiotoxin sequences provide a promising starting point for the development of toxin-inspired AMPs.

Using this rationale, we implemented two computational pipelines, including AMPA-assisted sequence mining and consensus-based analysis integrated with machine learning/deep learning-based antimicrobial prediction tools to identify peptide candidates derived from CTX sequences. In summary, the dual strategy enabled the in silico prioritisation of a panel of CTX-inspired peptides that were subsequently evaluated experimentally. Among these candidates, peptides derived from a region corresponding to *Naja pallida* CTX emerged as the most promising, indicating that region as the active core sequence. Peptides mimicking other regions of the CTX were not able to kill the bacterial species tested. Notably, CTX-p5 displayed the highest antibacterial activity within the peptide library, exhibited an ability to disrupt bacterial membranes, and adopted a defined α-helical conformation in SDS buffer, as demonstrated by CD studies. Although several peptides displayed increased α-helical signatures in SDS or TFE, these environments do not fully reproduce the physicochemical complexity of biological lipid membranes and are known to stabilise helical conformations artificially^41^. Consequently, peptide helicity in native bacterial membranes may be lower than that observed in detergent or organic solvent systems. This limitation may partially explain the discrepancy between AlphaFold structural predictions, membrane-mimicking CD measurements, and the relatively weak antimicrobial activity observed experimentally. In addition, antimicrobial and membrane-disrupting peptides may act through multiple mechanisms that do not necessarily depend on stable α-helical conformations^42^. Consequently, the relationship between predicted structural propensity, experimentally observed secondary structure, and antibacterial activity is likely more complex than a direct association between helicity and membrane disruption.

CTX-p5 retains physicochemical features commonly associated with membrane-active AMPs^43,44^, including cationic character and amphipathic residue distribution, which interact with bacterial membrane components. However, our results also indicate that increasing the positive charge alone does not necessarily enhance antimicrobial activity, as illustrated by the reduced activity of the analogue CTX-p6, which contains additional charged residues but displays lower antibacterial potency. This observation suggests that antimicrobial activity depends on a balanced combination of charge, hydrophobicity, and spatial residue organisation, rather than charge density alone. Similar behaviour has been described for other AMPs whose membranolytic activity relies primarily on global physicochemical properties rather than the formation of a stable α-helical structure^44,45^.

Further analysis revealed that sequence truncation or modification of CTX-p5, including shorter analogues such as CTX-p6 and related derivatives, resulted in reduced antibacterial activity. These findings indicate that the antimicrobial effect observed for CTX-p5 is dependent on the specific sequence length and residue arrangement within this region of the cardiotoxin scaffold. Similar observations have been reported for other AMPs in which truncation disrupts the spatial balance between cationic residues and hydrophobic side chains, thereby impairing membrane interaction and reducing antimicrobial potency^46,47^. In addition to the loss of antibacterial activity, some sequence modifications also increased haemolytic activity, suggesting that relatively small alterations in peptide composition can significantly affect membrane selectivity between bacterial and mammalian cells. This phenomenon has been widely documented in AMP engineering, where changes in peptide length, hydrophobicity, or amphipathicity can alter the balance between antibacterial efficacy and host-cell toxicity^48,49^. Interestingly, CTX-p5 itself displayed lower haemolytic activity compared with its lipidated analogue (CTX-p7), indicating a more favourable balance between antibacterial activity and erythrocyte toxicity. Lipidation is frequently used to enhance peptide–membrane interactions and antimicrobial potency^50^; however, this chemical modification can also increase affinity for mammalian membranes, often leading to elevated haemolysis^51^. Similar effects have been reported for synthetic lipidated AMPs, where the introduction of hydrophobic moieties improves bacterial membrane binding but simultaneously reduces selectivity^52,53^. Importantly, these observations are also consistent with previous work demonstrating that CTX-derived fragments often possess limited intrinsic antimicrobial activity unless sequence optimisation is introduced^27^. In the study by Sala et al^27^., multiple amino acid substitutions were required to improve antibacterial potency of CTX-derived peptides. Together, these findings highlight the importance of maintaining an optimal balance between charge, hydrophobicity, and peptide length in the refinement and selection of membrane-active peptides, and reinforce the role of cardiotoxin-derived fragments as starting templates whose activity and selectivity may be further optimised through rational modifications that stabilise membrane-active conformations, particularly cyclisation and targeted amino acid substitutions. From this perspective, future optimisation of CTX-derived fragments should focus not only on increasing charge or hydrophobicity but also on stabilising membrane-active conformations. Cyclisation may be particularly favourable because it can constrain peptide geometry, reduce conformational flexibility, improve proteolytic stability, and enhance protection of backbone amide groups in membrane-associated states^54^. Such effects may favour deeper and more stable membrane insertion compared with flexible linear fragments^55^. By contrast, lipidation, as observed for CTX-p7, may increase membrane affinity but can also enhance erythrocyte disruption and reduce selectivity. PEGylation may improve solubility, circulation time, or pharmacokinetic behaviour^56^, but it is less clear that it would improve antibacterial potency of these short CTX-derived fragments. Therefore, future engineering should prioritise conformational constraint, selective hydrophobicity tuning, and targeted amino acid substitutions designed to stabilise amphipathic membrane-active structures while avoiding excessive nonspecific membrane disruption.

Despite the large number of candidates predicted by the computational tools employed in this study, only a limited number of peptide candidates displayed any measurable antibacterial activity. This observation highlights the current limitations of AI-based AMP prediction platforms in accurately identifying functional peptides from toxin-derived sequences. The prediction tools used here, including CAMP-R4^57^, AMP Scanner v2^32^, AMPlify^29^ and amPEPpy^25^, rely primarily on machine learning models trained on datasets of known AMPs, typically using features such as amino acid composition, physicochemical descriptors, and sequence motifs. While these approaches are effective for identifying peptides that share general characteristics with canonical AMPs^58,59^, they can also generate a high number of false-positive candidates^60,61^, particularly when applied to peptide fragments derived from larger toxin scaffolds. One contributing factor is that many prediction models emphasise global features such as cationic charge, hydrophobicity, and amphipathicity, which are common among AMPs but are not sufficient on their own to confer biological activity^62^. Furthermore, recent benchmarking studies have demonstrated that the reported performance of many AMP predictors may be inflated due to biases in the construction of benchmark datasets, particularly the selection of negative examples that differ substantially from true AMPs in length, composition, or biological context^63^. Similar limitations have been reported in other studies evaluating AMP prediction tools^50,64^. For example, systematic benchmarking analyses have shown that widely used predictors frequently misclassify peptides with favourable physicochemical properties as antimicrobial despite lacking experimental activity, highlighting the risk of high false-positive rates when screening novel peptide sources^63^. In particular, models trained predominantly on α-helical AMPs may perform poorly when evaluating peptides derived from toxin families with different structural frameworks, such as cardiotoxins or other venom proteins. In addition, most predictive algorithms do not explicitly account for context-dependent structural behaviour, membrane interactions, or peptide aggregation properties, all of which can strongly influence antimicrobial efficacy^65,66^. Consequently, peptides predicted to be antimicrobial based on sequence features alone may fail to adopt the appropriate conformation or membrane interactions required for biological activity. Another limitation of the computational workflow is that the prediction tools used in this study primarily estimate general antimicrobial potential rather than activity against specific bacterial species. Recent prediction platforms, including strain-oriented models available through DBAASP^35^, enable species-specific activity prediction against organisms such as *E. coli* or *S. aureus*, which may improve candidate prioritisation during peptide discovery. Similarly, structural prediction tools such as AlphaFold may have limited predictive accuracy for short linear peptides with high conformational flexibility. Several peptides predicted to adopt helical conformations displayed only weak or partial α-helical signatures experimentally, suggesting that computational structural propensity alone may not reliably predict membrane-active behaviour or antibacterial potency. Furthermore, membrane-active peptides frequently undergo environment-dependent conformational transitions that are influenced by membrane lipid composition. Since no current computational method, including AlphaFold, can reliably predict these membrane-bound conformations, theoretical structural predictions should be interpreted with caution when assessing antimicrobial peptide activity. Hence, these limitations underscore the importance of combining computational predictions with experimental validation when exploring toxin-derived peptides as potential antimicrobial candidates.

Interestingly, peptides derived from the consensus CTX scaffold did not display measurable antibacterial activity. Although consensus sequences capture residues conserved across toxin homologues, this approach may dilute local sequence features that are essential for biological function. In addition, effective AI-guided peptide discovery workflows often rely on the generation and screening of very large virtual peptide libraries comprising thousands or even hundreds of thousands of candidates prior to experimental prioritisation^67^. In the present study, the relatively small number of generated peptides and the use of consensus-derived templates may have limited the exploration of broader sequence diversity. Consensus sequence generation may also dilute local residue arrangements required for membrane interaction and biological activity, thereby reducing the likelihood of identifying highly active peptide fragments. Antimicrobial peptides often depend on precise arrangements of cationic and hydrophobic residues that enable amphipathic membrane interactions^68^. In the case of cardiotoxins, such functional motifs may occur within specific structural loops rather than across globally conserved regions of the protein. Consequently, averaging sequences into a consensus template may disrupt these local physicochemical patterns, reducing the likelihood of recovering active peptide fragments. In contrast, the region corresponding to CTX-p5 originates from a specific cardiotoxin sequence and preserves the native residue distribution required for membrane interaction, which may explain its retained antimicrobial activity. Cardiotoxins adopt a rigid three-finger β-sheet architecture in which membrane-interacting residues are spatially organised within exposed loop regions^23,69^. Peptides derived from these loops may retain membrane-active properties, whereas fragments extracted from other regions of the scaffold may lack the structural determinants required for lipid interaction.

Comparison with previous studies on full-length cardiotoxins and cardiotoxin-like proteins provides a possible explanation for the modest activity of the CTX-derived fragments described here. Full-length CTXs retain the native three-finger scaffold, in which membrane-binding residues are spatially organised at the exposed loop extremities^22,69^, and this preserved architecture can support stronger membrane interaction and species-dependent antibacterial activity. For example, full-length cobra CTXs or CTX-like proteins have been reported to display antibacterial activity^39^. In contrast, short linear fragments derived from CTX sequences may lose the spatial organisation imposed by the native scaffold and may display reduced or unstable membrane-associated secondary structure. This may limit productive insertion into bacterial membranes and partly explain the relatively weak MIC values observed in the present study. Such concentrations are higher than those typically associated with clinically advanced antimicrobial peptides and therefore currently limit the translational applicability of these candidates as direct therapeutic agents. These findings emphasise the need for further optimisation strategies aimed at improving antibacterial potency.

Notably, the in silico predictions of haemolytic potential showed a closer agreement with the experimental data than the antimicrobial activity predictions. Most peptides predicted to exhibit low haemolytic potential indeed produced limited erythrocyte membrane disruption in vitro, whereas pepitdes which displayed stronger haemolytic activity experimentally were also predicted to have higher toxicity scores. This improved correspondence may partly reflect the relatively simple and homogeneous nature of red blood cells, which represent a single mammalian cell type with a well-defined membrane composition^70^. In contrast, bacteria exhibit substantial diversity in membrane architecture and lipid composition between species, including differences in outer membranes, peptidoglycan layers, and phospholipid distributions^66,71,72^. Such variability can influence peptide–membrane interactions and antimicrobial efficacy, making antibacterial activity more difficult to predict accurately from sequence-based computational models^73^.

Importantly, cardiotoxin-derived fragments represent only one class of toxin-inspired antimicrobial templates. Other venom-derived peptide families, including naturally linear amphipathic peptides from insect or spider venoms, may provide particularly favourable scaffolds for antimicrobial development due to their strong membrane activity, inducible α-helical propensity in lipid environments, and in some cases selective interactions with bacterial membrane lipids. Such peptides often possess physicochemical features that favour stable membrane insertion and potent antibacterial activity while retaining relatively simple linear architectures amenable to engineering^74^. Integrating knowledge of lipid-selective motifs, membrane-associated structural behaviour, and toxin evolution may therefore improve future AI-guided antimicrobial peptide discovery strategies. Future studies may also benefit from the inclusion of additional peptide controls unrelated in sequence origin and biological function, including scrambled peptides or peptides derived from unrelated protein families with comparable physicochemical characteristics. Such comparisons could provide further insight into the extent to which the observed antimicrobial and membranolytic effects arise from sequence-specific structural features versus more general properties associated with peptide charge, amphiphilicity, or hydrophobicity.

Taken together, this work provides a proof-of-principle that toxin sequence mining combined with computational prediction can generate membrane-active peptide candidates from venom toxins. In silico analysis enabled the rapid identification of antibacterial CTX-derived encrypted regions, leading to the prioritisation of a library of CTX-inspired peptides. At the same time, our results highlight the importance of critically evaluating and improving machine learning-based prioritisation strategies. Experimental validation revealed that only a small portion of these candidates showed measurable antibacterial effects, with CTX-p5 emerging as a moderately active membrane-disrupting antibacterial peptide. Snake venoms contain a vast diversity of toxins targeting multiple cellular processes beyond membranes, including ion channels, receptors, and enzymatic pathways. Expanding toxin-inspired discovery efforts to these diverse molecular targets may open new opportunities for biomedical applications. Importantly, reporting moderate activities and unsuccessful predictions is equally valuable for improving computational models and guiding future discovery efforts. Building on these initial templates, further rational optimisation of CTX-derived peptides may enable the development of more potent and selective antimicrobial agents. In addition, continued integration of toxin sequence mining with improved computational models and experimental validation may expand opportunities for toxin-inspired peptide drug discovery. In this context, other toxin scaffolds are equally suitable for generating potent linear antimicrobial peptides. Venom-derived peptide families that naturally exhibit strong membrane activity, inducible α-helicity in lipid environments, or selective lipid-binding motifs, such as peptides from insect or spider venoms, may represent particularly promising candidates for future antimicrobial development. Combining AI-guided sequence mining with mechanistic understanding of membrane interactions and structural stabilisation may facilitate the discovery of more potent and selective antimicrobial peptides capable of targeting antibiotic-resistant bacteria.

## Methods

### Sequence mining and computational prioritisation of CTX-derived peptides

A total of 14 peptides were selected based on cardiotoxins/cytotoxins derived from *Naja* species using two complementary strategies. In the first approach, eight CTX proteins were obtained from UniProtKB/Swiss-Prot and UniProtKB/TrEMBL, focusing on sequences with highly conserved domains, including a cardiotoxin from *N. atra* previously reported as a potential source of antimicrobial peptides^27^. These proteins (~60 amino acids) were analysed using the AMPA algorithm^28^ to identify putative antimicrobial regions. AMPA software was used as a deterministic sequence-analysis tool to identify physicochemically favourable antimicrobial regions rather than as an artificial intelligence-based peptide design platform. This strategy yielded seven candidate peptides named as CTX-p1, CTX-p2, CTX-p3, CTX-p4, CTX-p5, CTX-p8 and CTX-p9, all derived from naturally occurring sequences. Two additional modified peptides (CTX-p6 and CTX-p7) were designed based on CTX-p5, incorporating lysine substitution and a palmitoyl tail, respectively. In the second approach, a data-mining strategy was applied using UniProtKB. A refined search (keywords: “cytotoxin”; taxonomy: *Naja* spp.; review status: Swiss-Prot; annotation level: protein) yielded 92 sequences. Multiple sequence alignment (MSA) was conducted using Clustal Omega, and the results were visualised and curated using Jalview (version 2.11.5.0). A consensus sequence was subsequently generated in Jalview based on residue frequency conservation across aligned positions, where the most frequently occurring amino acid at each position was selected as the consensus residue. This consensus sequence was then used as a template for rational peptide design of five additional peptides (CTX-p10, CTX-p11, CTX-p12, CTX-p13 and CTX-p14). Following peptide identification through sequence mining or consensus analysis, candidate peptides were subsequently prioritised using machine learning-based AMP prediction tools, including CAMP-R4^30^, AMP Scanner v2^32^, AMPlify^29^ and amPEPpy^31^, as well as haemolytic prediction platforms such as HemoPI^33^ and HLPpred-Fuse^34^ to assess antimicrobial potential and haemolytic profiles. In summary, machine learning/deep-learning-based AMP prediction tools were used for peptide prioritisation and antimicrobial probability assessment, whereas peptide generation itself was primarily based on deterministic sequence-analysis and computational extraction of toxin-derived regions or consensus-derived peptide strategy. The antimicrobial prediction tools employed in this study are based on distinct machine learning and deep learning strategies. CAMP-R4 incorporates multiple classifiers, including support vector machine (SVM), random forest (RF), artificial neural network (ANN), and discriminant analysis algorithms trained on experimentally validated AMP datasets^30^. AMP Scanner v2 employs deep neural network architectures for AMP classification^32^. AMPlify uses an attentive deep learning framework based on bidirectional long short-term memory (Bi-LSTM) networks combined with attention mechanisms^29^, whereas amPEPpy employs a random forest classifier trained using sequence-derived physicochemical descriptors^31^. The haemolytic prediction platforms also rely on machine learning-based approaches. HemoPI uses machine learning models trained on experimentally validated haemolytic peptides and incorporates sequence-derived physicochemical descriptors and support vector machine-based prediction approaches^33^. HLPpred-Fuse employs a fused-feature prediction framework integrating multiple peptide descriptors with ensemble classification algorithms^34^. Default parameters and prediction settings recommended by each platform were used throughout the study.

### Synthesis and identification of CTX-derived peptides

Peptides were synthesised by solid-phase peptide synthesis using standard Fmoc chemistry and obtained from Peptide Protein Research Ltd. (Hampshire, UK). In the case of CTX-p7, for C-terminal lipidation, the peptide was first cleaved from the resin while retaining all side-chain protecting groups. Hexadecylamine was subsequently coupled to the peptide C-terminus in solution using standard peptide coupling chemistry, resulting in the formation of an amide bond between the peptide carboxyl terminus and hexadecylamine. Following successful coupling, side-chain protecting groups were removed under standard deprotection conditions. Purity was assessed by high-performance liquid chromatography (HPLC), and molecular masses were confirmed by mass spectrometry (MS) as described previously^50^. All peptides displayed a purity ≥95% (Fig. S5 and Table S1).

### Structural characterisation of CTX-inspired peptides by computational modelling and circular dichroism

The three-dimensional structures of the CTX-inspired peptides were predicted using the AlphaFold 2 protein structure prediction platform (https://alphafold.com/). The resulting models were visualised and analysed to evaluate the structural features of the peptides. In addition, helical wheel projections (https://heliquest.ipmc.cnrs.fr/) were generated to examine the distribution of hydrophobic and hydrophilic residues and assess the amphipathic nature of the peptide sequences.

Experimental secondary structure analysis was performed using circular dichroism (CD) spectroscopy. Spectra were recorded on a Chirascan spectropolarimeter (Applied Photophysics, UK) using a 0.1 mm pathlength quartz cuvette. Measurements were acquired at 25 °C over a wavelength range of 180–250 nm, with each spectrum representing the average of three accumulations. A buffer baseline was recorded under identical conditions and subtracted from all spectra. Peptides were prepared at 1 mM aqueous solution, 30 mM sodium dodecyl sulfate (SDS), or 40% (v/v) trifluoroethanol (TFE). SDS and TFE were employed as membrane-mimicking environments to evaluate inducible secondary structure formation, although these systems do not fully replicate the complexity of biological lipid bilayers. CD data were converted to mean residue ellipticity ([θ], deg·cm²·dmol⁻¹) using Chirascan ProData Viewer (Applied Photophysics). High-tension (HT) voltage values remained below 600 V throughout the recorded spectral range throughout the measurements.

Secondary-structure estimation was performed by constrained spectral deconvolution analysis of the normalised CD spectra using reference basis spectra corresponding to α-helix, β-sheet, turn, and random coil conformations. Experimental spectra were fitted over the 195–250 nm wavelength range using constrained least-squares optimisation, and the relative contributions of each structural component were estimated. The quality of spectral fitting was evaluated by comparison between experimental and reconstructed spectra. The data were carried out using the SELCON3, CONTINLL and CDSSTR algorithms available through the DichroWebGit server.

### Antimicrobial screening

The antibacterial activity of the CTX-inspired peptides was evaluated by determining the minimum inhibitory concentration (MIC) using the standard broth microdilution method. A panel of Gram-negative bacteria, including *Escherichia coli* (ATCC 25922 and enterohaemorrhagic O157:H7 str. EDL933) and *Pseudomonas aeruginosa* (PAO1), as well as Gram-positive bacteria including *Staphylococcus aureus* (ATCC 12600 and methicillin-resistant *S. aureus* NCTC 13656 (MRSA)), was used for in vitro screening. Overnight bacterial cultures grown in Lysogeny broth (LB) from single colonies were diluted to approximately 5 × 10^5^ CFU mL⁻¹ in Mueller–Hinton broth (MHB). Peptides were serially diluted two-fold in 96-well microplates to final concentrations ranging from 0 to 500 μM and incubated with bacterial suspensions overnight at 37 °C. Bacterial growth was monitored spectrophotometrically using a Tecan Spark (O.D._600_), and the MIC was defined as the lowest peptide concentration that completely inhibited bacterial growth. Experiments were independently repeated three times, with each assay performed in triplicate.

### Atomic force microscopy

The effects of CTX-p5 on bacterial cell morphology were examined using atomic force microscopy (AFM). Mid-log phase *E. coli* cultures (5 × 10^6^ CFU mL⁻¹) were incubated with CTX-p5 at twice the MIC in MHB for 3 h at 37 °C. Cells were collected, resuspended in phosphate-buffered saline (PBS), and fixed overnight with 2.5% glutaraldehyde. After fixation, samples were washed three times with water and deposited onto muscovite mica mounted on metal stubs. AFM imaging was performed using an Asylum Research Cypher S microscope in air tapping mode with a scan size of 30 µm, 512 points per line, and a scan rate of 0.75 Hz.

### Fluorescence staining and microscopy analysis of bacterial membrane permeability

To further investigate the membrane-disrupting effects of the most active peptide, CTX-p5, bacterial viability and membrane integrity were analysed using fluorescence-based staining combined with flow cytometry and microscopy. Mid-log phase *E. coli* cultures were adjusted to approximately 5 × 10⁶ CFU mL⁻¹ in MHB and exposed to CTX-p5 at concentrations corresponding to one- and two-fold MIC for 1 h at 37 °C. Following incubation, bacterial suspensions were washed three times and resuspended in 0.85% NaCl solution. Cells were subsequently stained using a Bacteria Live/Dead Staining kit (Promokine) according to the manufacturer’s instructions and incubated for 15 min at room temperature. Untreated bacteria and cells treated with 1% Triton X-100 served as negative and positive controls, respectively. Fluorescence signals were measured using a CytoFLEX S Flow Cytometry (Beckman Coulter), and data were acquired from approximately 10,000 events per sample.

Membrane disruption was further visualised using fluorescence microscopy. *E. coli* cultures (mid-logarithmic growth phase) were incubated with CTX-p5 at the MIC for 1 h at 37 °C. Bacterial cells were collected by centrifugation, resuspended in 0.85% NaCl, and stained with DMAO and EthD-III (Promokine). A small volume of the stained suspension was placed onto microscope slides and examined using a Nikon Eclipse Ti inverted fluorescence microscope. All assays were performed independently in three separate experiments, each conducted in triplicate.

### Outer membrane permeabilisation assay

Mechanistic studies were performed using the most active peptide, CTX-p5. The ability of CTX-p5 to permeabilize the bacterial outer membrane was evaluated using the fluorescent probe N-phenyl-1-naphthylamine (NPN), which exhibits increased fluorescence in hydrophobic environments. Overnight cultures of *E. coli* were adjusted to an OD_600_ of 0.5 in 5 mM HEPES buffer (pH 7.4) supplemented with 5 mM glucose and transferred to black-bottom 96-well plates (Greiner). NPN was added to a final concentration of 10 µM and incubated for 15 min at 37 °C. CTX-p5 was then added at twice the MIC, and fluorescence intensity was monitored for 1 h at 37 °C using a SpectraMax plate reader (excitation 350 nm; emission 420 nm). Independent experiments were repeated three times.

### Membrane depolarisation assay

Inner membrane bacteria depolarisation by CTX-p5 was assessed using the membrane potential–sensitive dye 3,3′-dipropylthiadicarbocyanine iodide [DiSC₃(5)]. *E. coli* cells adjusted to OD_600_ = 0.5 in HEPES buffer (pH 7.4) supplemented with 5 mM glucose and 0.1 M KCl were incubated with 0.5 µM DiSC₃(5) for 15 min to allow for fluorescence quenching. Then CTX-p5 was subsequently added at twice the MIC, and fluorescence changes were monitored in raried outeal time using a SpectraMax microplate reader (λex = 622 nm; λem = 670 nm). Independent experiments were repeated three times.

### Assessment of erythrocyte membrane lysis by CTX-inspired peptides

The haemolytic activity of the CTX-inspired peptides was evaluated using donated human red blood cells (hRBCs) obtained from three healthy volunteer donors in accordance with the protocol approved by the University of Reading Research Ethics Committee (UREC 17/17). Fresh erythrocytes were washed three times with phosphate-buffered saline (PBS) and resuspended in the same buffer to obtain a final suspension of 0.5% (v/v). Peptide solutions were prepared in water and tested at final concentrations of 62.5, 125, 250, 500, and 1000 µM in flat-bottom 96-well microplates. Equal volumes of peptide solution and erythrocyte suspension were combined and incubated for 1 h at 37 °C. After incubation, samples were centrifuged at 1000 × *g* for 10 min at 4 °C to pellet intact erythrocytes. Sixty microlietrs from the supernatants were carefully transferred to new 96-well plates, and haemoglobin release was quantified by measuring absorbance at 414 nm using a Tecan Spark plate reader. PBS and 1% Triton X-100 were used as negative and positive controls, representing baseline and complete haemolysis, respectively. The percentage of haemolysis was determined by comparing haemoglobin release from peptide-treated samples with the control conditions. All experiments were performed in triplicate.

### Statistical analysis

All data are presented as mean ± s.d. Statistical analyses were performed using GraphPad Prism 8. Comparisons between control (bacteria only) and peptide-treated groups were carried out using one-way analysis of variance (ANOVA) followed by Tukey’s multiple comparisons test. Statistical significance was defined as *P* < 0.001.

## Supplementary information

Supplementary Information

## Data Availability

All data supporting the findings of the current study are available within the article and its supplementary information files. Peptide sequences analysed in this study were retrieved from the UniProtKB/Swiss-Prot database (accessed January 2026).
