# Supplementary material for: Harnessing snake venom cardiotoxins for antimicrobial peptide discovery
Source: NPJ Drug Discov. 2026 Sep 16;3:40. doi: 10.1038/s44386-026-00073-2 (PMC13582888; doi:10.1038/s44386-026-00073-2)
Supplement: Supplementary file 1 [file 44386_2026_73_MOESM1_ESM.pdf]

Supplementary Information

Harnessing snake venom cardiotoxins for antimicrobial peptide discovery

Bruno Mendes[1], José R. Almeida[2], Valeria Castelletto[3], Ian W. Hamley[3] and Glyn Barrett[1]*

[1]School of Biological Sciences, University of Reading, Reading, RG6 6UB, United Kingdom

[2] School of Pharmacy, University of Reading, Reading RG6 6UB, United Kingdom

[3]School of Chemistry, Pharmacy and Food Biosciences, University of Reading, Reading, RG6 6AD United Kingdom

*Corresponding author: Glyn Barrett, glyn.barrett@reading.ac.uk

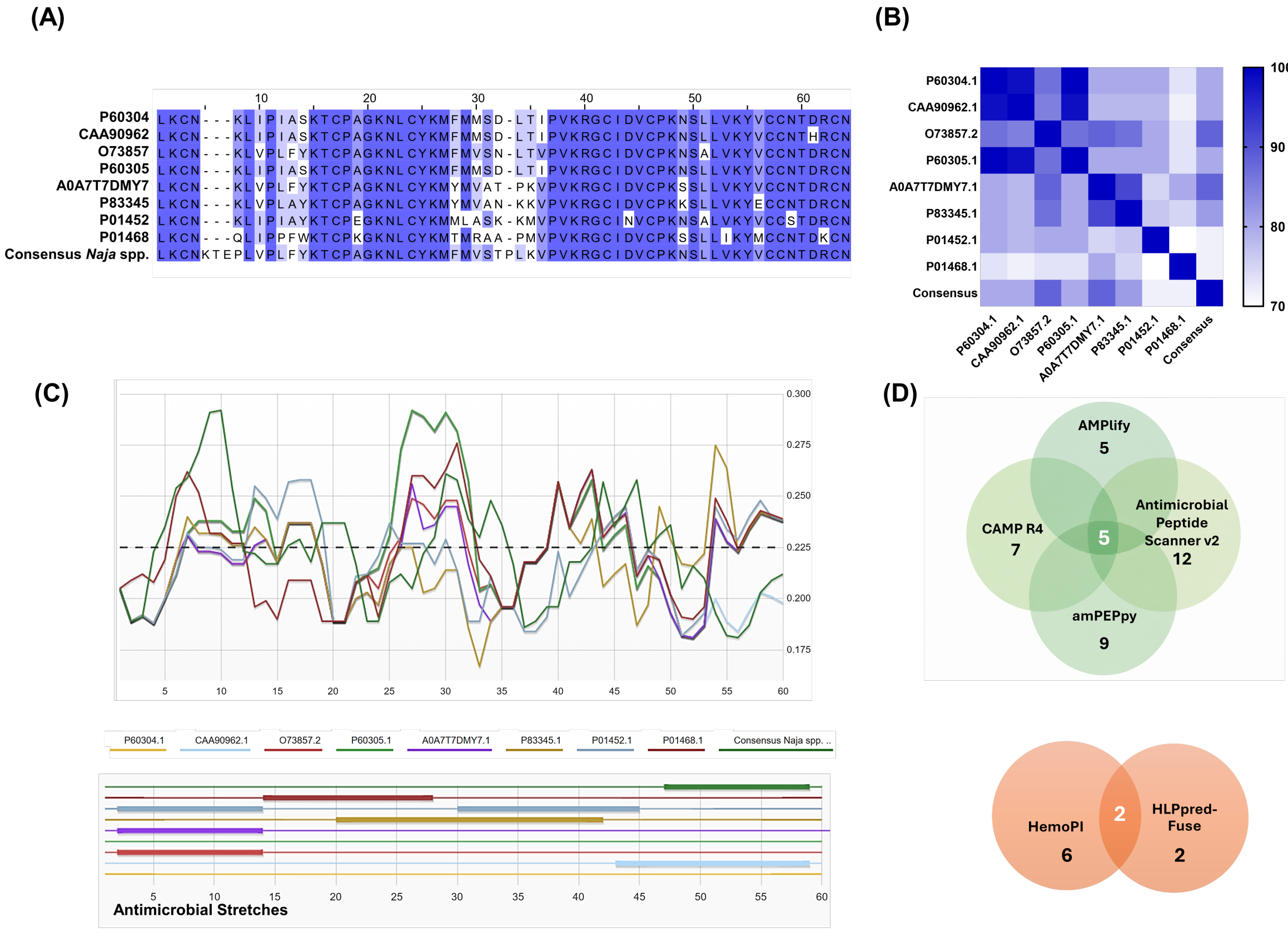

Figure S1. Computational sequence mining of cardiotoxin family sequences from eight *Naja* species to identify encrypted antimicrobial peptides. (A) Multiple sequence alignment showing highly conserved motifs across species. Consensus-*Naja spp.* represents the consensus sequence generated from a multiple sequence alignment comprising 92 CTX/CLBP-related sequences from *Naja* species. To evaluate sequence conservation and variation, we compared eight sequences from different *Naja* species against this consensus sequence. These eight sequences were used to prioritise the peptides in the first computational approach. **(B)** The heatmap illustrates pairwise sequence similarity relationships among the toxins selected for AMPA-based sequence scanning analysis, together with the derived consensus sequence. **(C)** The upper panel displays antimicrobial propensity profiles generated by AMPA across the aligned toxin sequences, with the dashed horizontal line representing the AMPA

threshold used for antimicrobial region prediction. The X-axis represents the amino acid position along the protein sequence, whereas the Y-axis indicates the antimicrobial propensity score calculated by AMPA at each position. The lower panel indicates the antimicrobial stretches predicted by AMPA for each analysed sequence, represented as bold coloured segments. Seven antimicrobial stretches were identified across the selected toxin sequences, with one additional stretch identified in the consensus-derived sequence. Some predicted stretches partially overlap within a shared sequence region among multiple CTXs. **(D)** Encrypted antimicrobial peptides (highlighted in green Venn diagram) showing five candidates consistently predicted by AMPlify, CAMP R4, amPEPpy and Antimicrobial Peptide Scanner. Red Venn diagram illustrates discrepancies in haemolytic predictions between HemoPI and HLPpred-Fuse, with two peptides predicted as haemolytic by both algorithms.

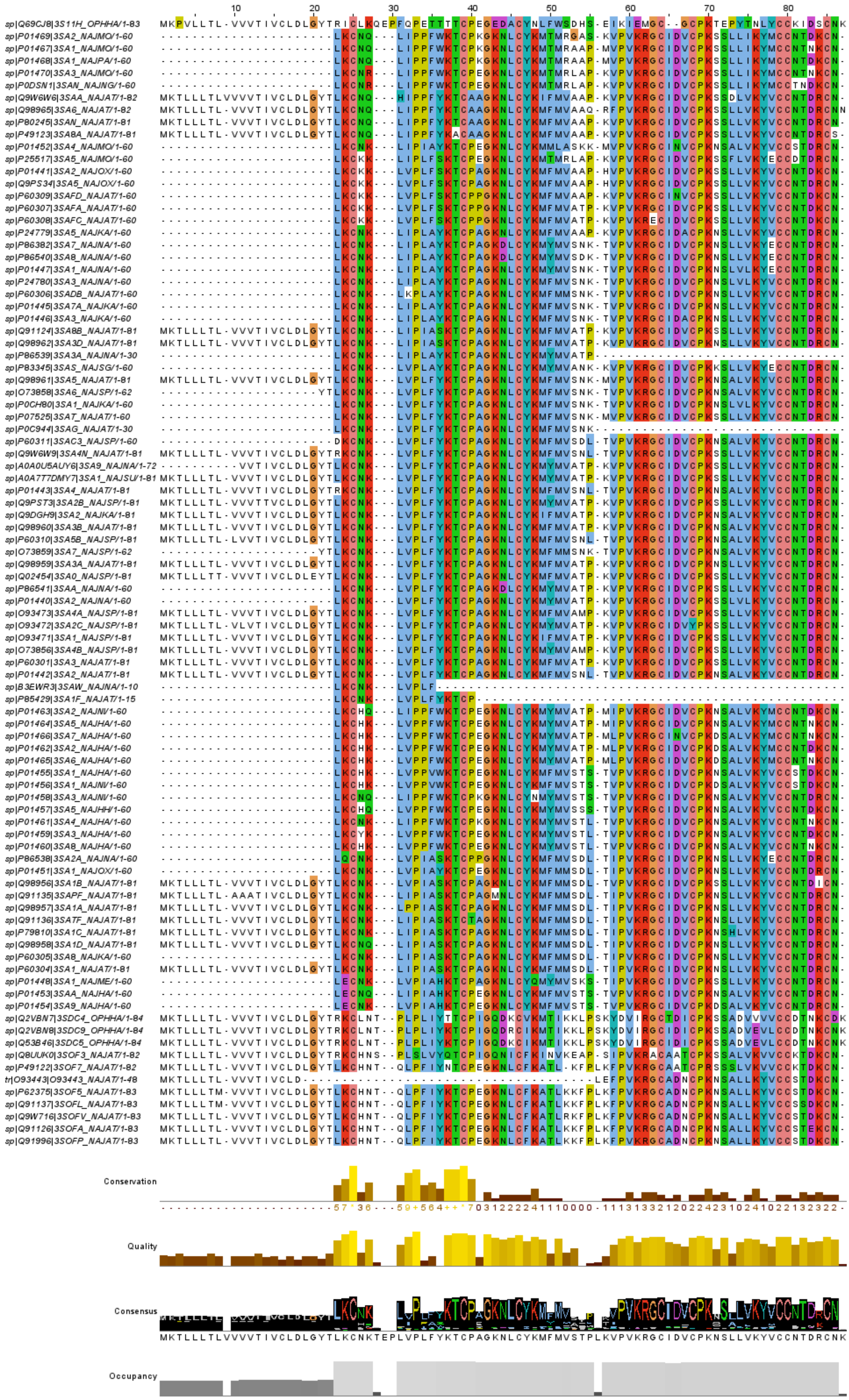

Conservation
Quality
Consensus
MKTLLLTLVVVVTIVCLDLGYTLKCNKTEPLVPLFYKTCPAGKNLCYKMFMVSTPLKVPVKRGCIDVCPKNSLLVKYVCCNTDRCNK
Occupancy

Figure S2. Clustal-based multiple sequence alignment of cardiotoxins from *Naja* species for antimicrobial peptide scaffold identification. The alignment reveals conserved cysteine-rich regions characteristic of the cardiotoxin protein**.** A consensus sequence, generated from 87 cardiotoxin sequences using Jalview 2.11.5.1, was used as the basis for a second approach to identify potential antimicrobial peptides. Conserved motifs within the mature toxin region guided the selection of candidate antimicrobial regions. The 'Conservation' histogram indicates residue conservation across aligned positions based on physicochemical similarity and amino acid conservation patterns. The 'Quality' score reflects the reliability and consistency of the alignment at each position, with higher values indicating greater alignment confidence. The 'Consensus' track displays the consensus amino acid sequence derived from the alignment. The 'Occupancy' track represents the proportion of sequences containing a residue at each alignment position, thereby indicating the extent of gap occurrence across the alignment.

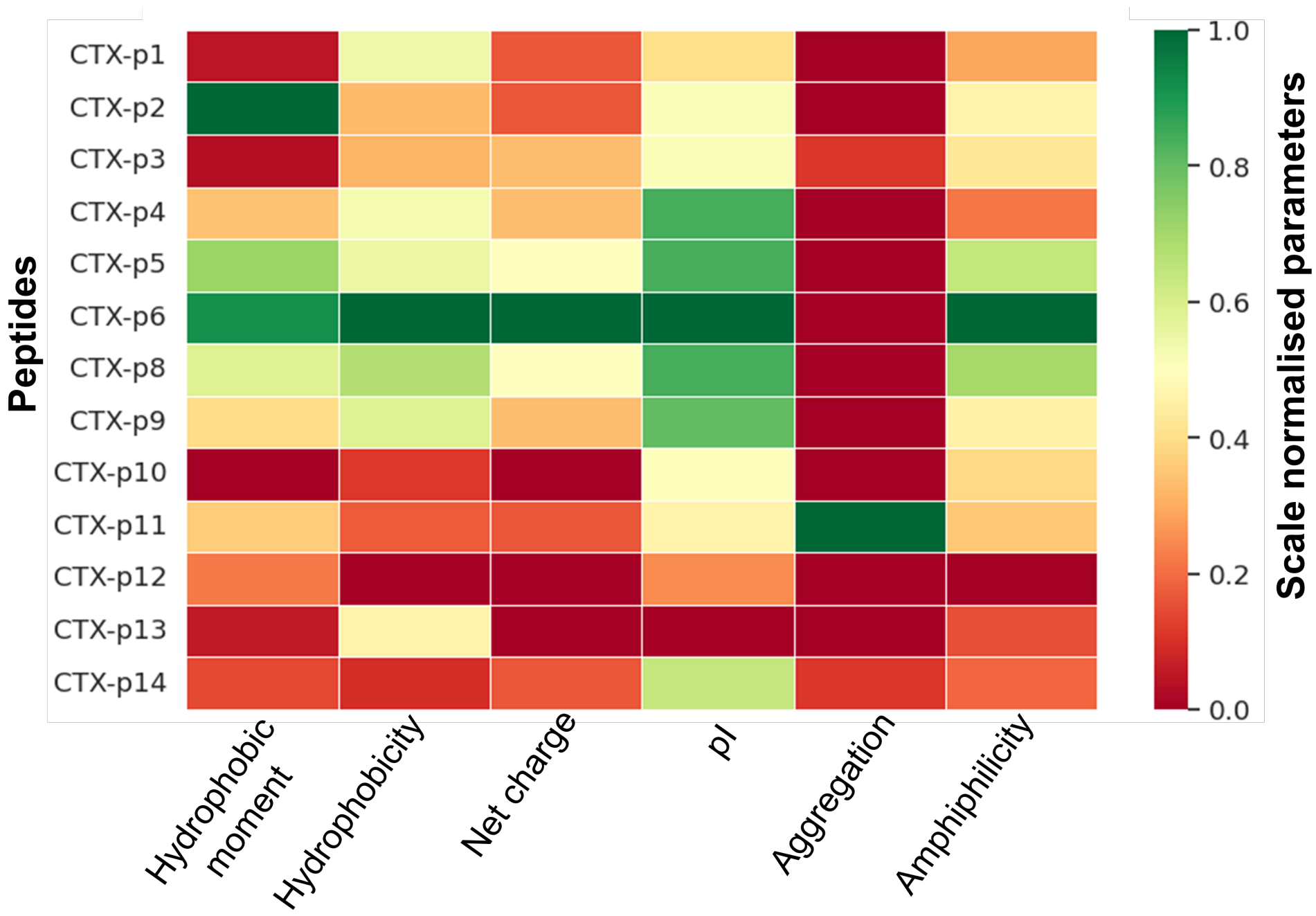

Figure S3. Physicochemical properties of CTX-derived peptides as a normalised heatmap. Distinct profiles across candidates were observed using the Database of Antimicrobial Activity and Structure of Peptides (DBAASP). Values were normalised to a scale ranging from 0 to 1, where red indicates lower values and green indicates higher values for a given property. The analysis reveals considerable variation among CTX peptides, highlighting differences in their physicochemical profiles CTX-p6 exhibits relatively high values across multiple properties, whereas CTX-p10, CTX-p12, and CTX-p13 display generally lower values. Certain properties show greater variability among peptides, suggesting distinct structural and functional characteristics within the CTX peptide dataset.

(A)

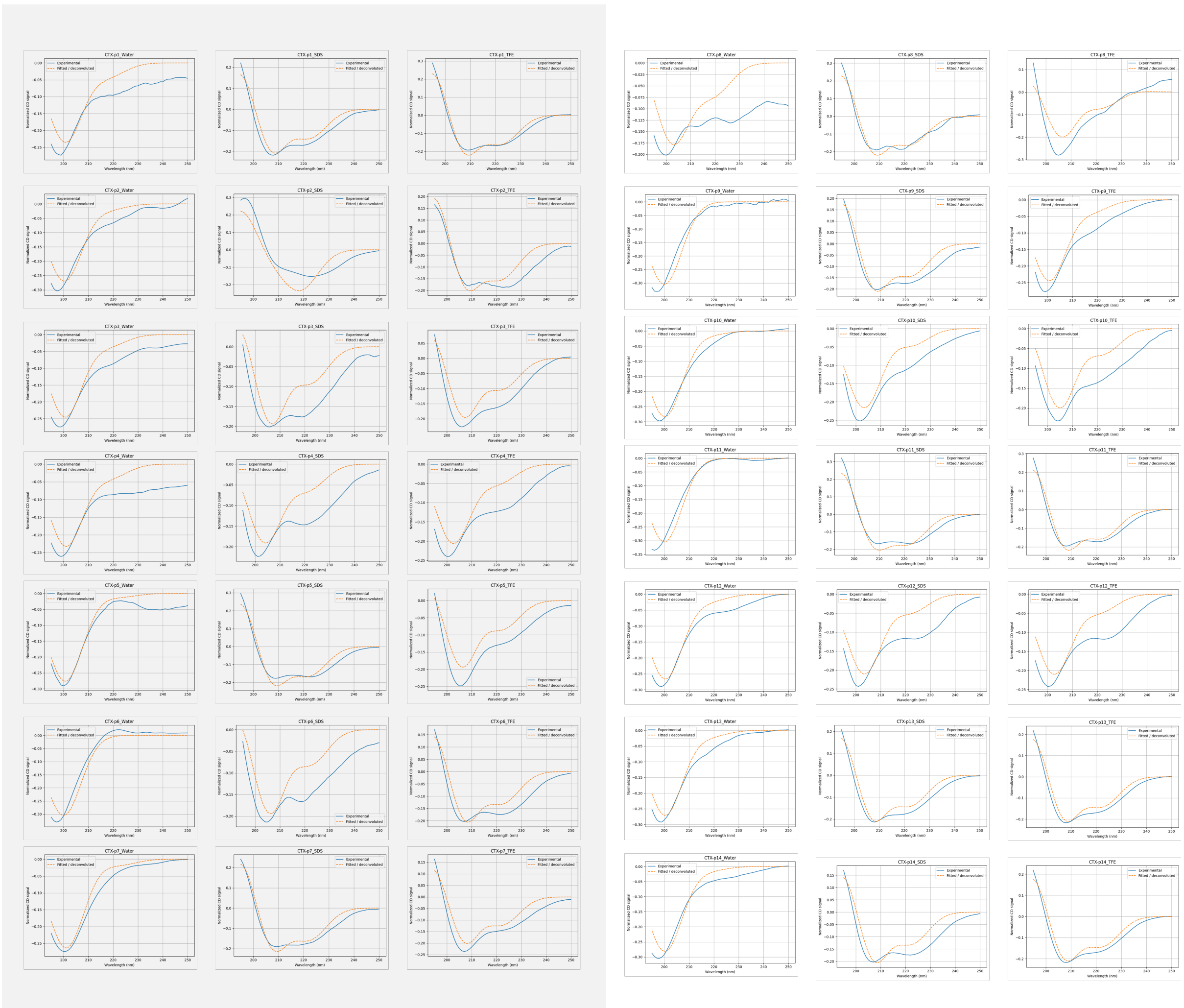

(B)

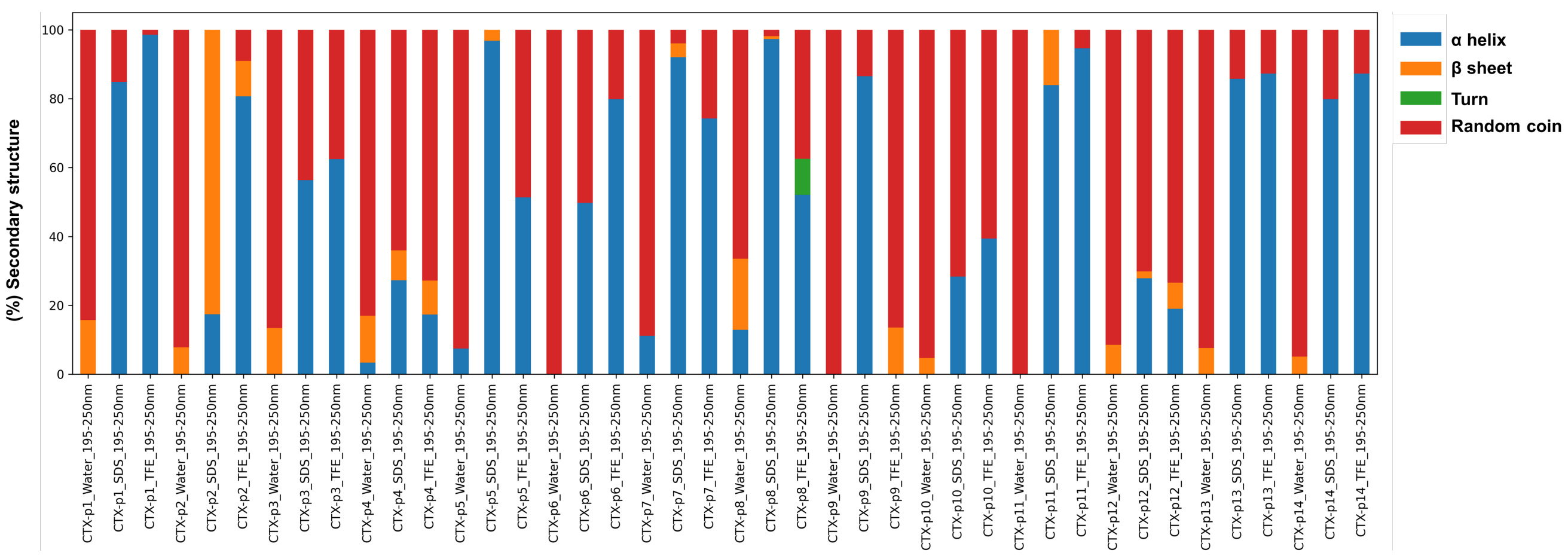

Figure S4. Secondary-structure deconvolution of CTX peptides under different solvent conditions (Water, SDS, and TFE) derived from circular dichroism (CD) spectra. **(A)** Experimental spectra (blue lines) and fitted/deconvoluted spectra (orange lines) are shown for each CTX peptides under the indicated conditions. Secondary-structure estimation was performed using constrained spectral

deconvolution of normalized CD spectra over the 195–250 nm. **(B)** Estimated relative contributions of α-helix, β-sheet, turn, and random coil structures. Membrane-mimetic environments, particularly SDS and TFE, promoted increased α-helical propensity compared with aqueous solution.

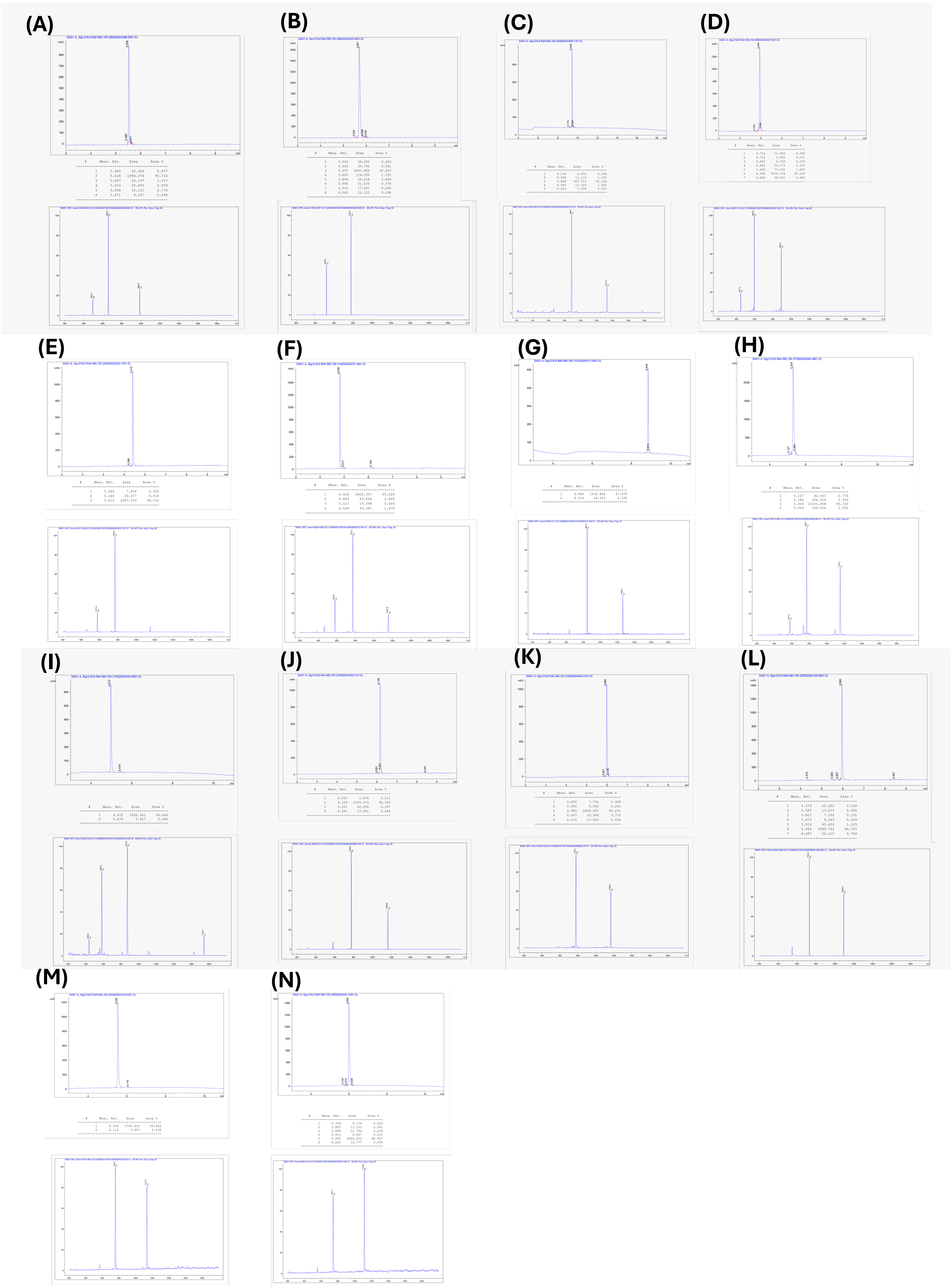

Figure S5. Purity determination and characterization of synthetic peptide by HPLC and mass spectrometry, respectively. Peptide profiles of (A) CTX-p1, (B) CTX-p2, (C) CTX-p3, (D) CTX-p4, (E) CTX-p5, (F) CTX-p6, (G) CTX-p7, (H) CTX-p8, (I) CTX-p9, (J) CTX-p10, (K) CTX-p11 and (L) CTX-p12, (M) CTX-p13, (N) CTX-p14 showing up to 95% purity levels. The peaks of retention times and the areas representing purity levels are highlighted in the upper graphs. The elution of synthetic

peptides was evaluated using a Kinetex XB-C18 column (100 Å, 4.6 x 50 mm) with a gradient ranging from 10% to 90% acetonitrile over 10 min. Bottom graphs show mass-to-charge ratio ions of products confirmed by mass spectrometry.

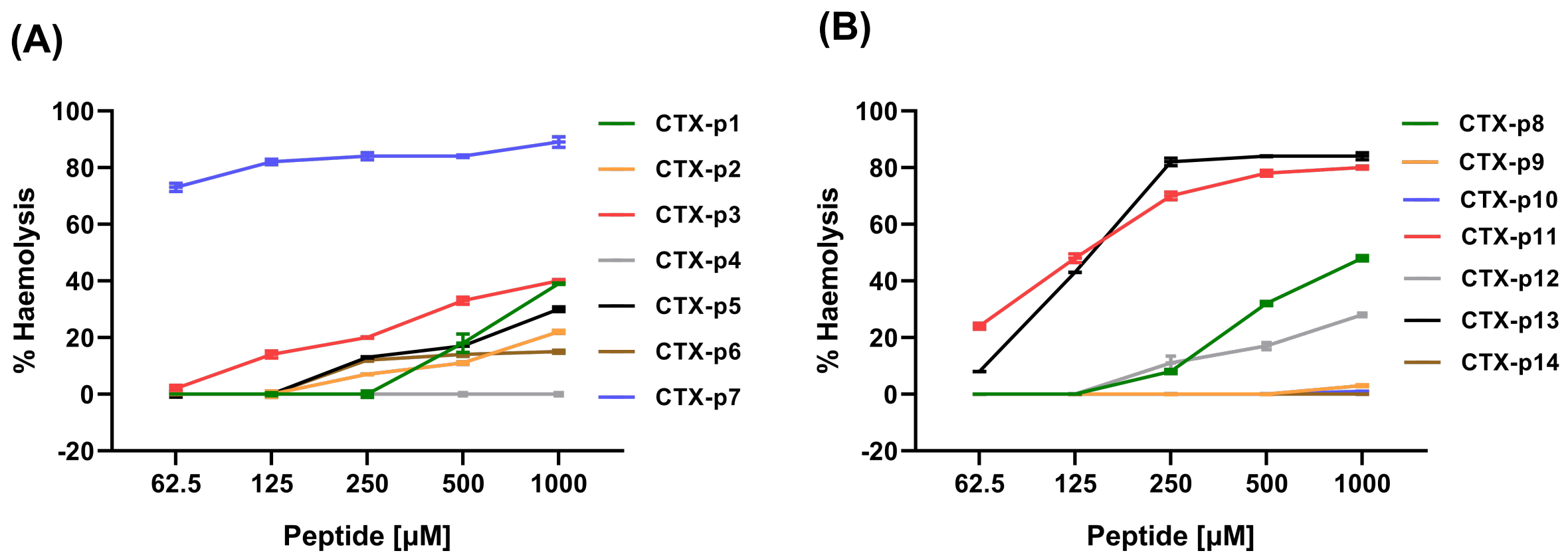

Figure S6. Haemolysis profiles of CTX-based peptides at concentrations of 62.5, 125, 250, 500, and 1000 µM. (A) Haemolytic activity of CTX-p1 to CTX-p7. **(B)** Haemolytic activity of CTX-p8 to CTX-p14. Data are presented as percentage haemolysis relative to the positive control. Values are expressed as mean ± SD from three independent experiments.

Table S1. Sequence and molecular mass of antimicrobial peptide candidates.

Peptide	Sequence	Molecular mass (Da)
**CTX-p1**	PKNSLLVKYVCCNTHRC	1977.383
**CTX-p2**	KCNKLVPLFYKTC	1555.948
**CTX-p3**	LCYKMYMVANKKVPVKRGCIDVC	2661.355
**CTX-p4**	KMVPVKRGCINVCPKN	1785.253
**CTX-p5**	FWKTCPKGKNLCYKMTMRA	2305.858
**CTX-p6**	FWKTCPKGKKLCYKKTKRA	2313.883
**CTX-p7**	FWKTCPKGKNLCYKMTMRA-NH-Palmitoyl	2530.287
**CTX-p8**	WKTCPKGKNLCYKMTMRA	2158.682
**CTX-p9**	CPKGKNLCYKMTMRA	1743.191
**CTX-p10**	LGYTLKCNKTEPLVPLFYKT	2327.793
**CTX-p11**	FYKTCPAGKNLCYKMFMVST	2331.846
**CTX-p12**	FMVSTPLKVPVKRGCIDVCP	2188.727
**CTX-p13**	VCPKNSLLVKYVCCNTDRCN	2271.709
**CTX-p14**	CPAGKNLCYKMFMVSTPLKV	2229.798
